# A New Clamp

**Published:** 1884-01

**Authors:** 


### A New Clamp.

I have used for several months past a rubber dam clamp, devised and constructed by Dr. J. P. Carmichael, of Milwaukee. It is a double clamp, the jaws of which consist of ten small steel plates, so hinged together as to make it adjustable, with a large range of adaptation, so that it fits accurately upon a tooth of any shape. It comes nearer being a universal clamp than anything yet presented to the profession. About three of these clamps, varying in size, would meet all cases so far as the shape and size of the teeth are concerned. The experience of the last six months in the use of this little appliance fully justifies the recommendation to every operator to try it. It is invaluable in many cases, and, perhaps, comes as nearly being universal in its application as could well be imagined. It is obtainable of dealers in dental supplies.

